# Machine Learning-Based Blood RNA Signature for Diagnosis of Autism Spectrum Disorder

**DOI:** 10.3390/ijms24032082

**Published:** 2023-01-20

**Authors:** Irena Voinsky, Oleg Y. Fridland, Adi Aran, Richard E. Frye, David Gurwitz

**Affiliations:** 1 Department of Human Molecular Genetics and Biochemistry, Faculty of Medicine, Tel Aviv University, Tel Aviv 69978, Israel; 2 Independent Researcher, Tel Aviv 69978, Israel; 3 Shaare Zedek Medical Center, Jerusalem 91031, Israel; 4 Obesity and Metabolism Laboratory, Institute for Drug Research, School of Pharmacy, Faculty of Medicine, The Hebrew University of Jerusalem, Jerusalem 91240, Israel; 5 Autism Discovery and Treatment Foundation, Phoenix, AZ 85050, USA; 6 Rossignol Medical Center, Phoenix, AZ 85050, USA; 7 Sagol School of Neuroscience, Tel Aviv University, Tel Aviv 69978, Israel

**Keywords:** machine learning, RNA biomarkers, blood RNA-sequencing, autism spectrum disorder (ASD)

## Abstract

Early diagnosis of autism spectrum disorder (ASD) is crucial for providing appropriate treatments and parental guidance from an early age. Yet, ASD diagnosis is a lengthy process, in part due to the lack of reliable biomarkers. We recently applied RNA-sequencing of peripheral blood samples from 73 American and Israeli children with ASD and 26 neurotypically developing (NT) children to identify 10 genes with dysregulated blood expression levels in children with ASD. Machine learning (ML) analyzes data by computerized analytical model building and may be applied to building diagnostic tools based on the optimization of large datasets. Here, we present several ML-generated models, based on RNA expression datasets collected during our recently published RNA-seq study, as tentative tools for ASD diagnosis. Using the random forest classifier, two of our proposed models yield an accuracy of 82% in distinguishing children with ASD and NT children. Our proof-of-concept study requires refinement and independent validation by studies with far larger cohorts of children with ASD and NT children and should thus be perceived as starting point for building more accurate ML-based tools. Eventually, such tools may potentially provide an unbiased means to support the early diagnosis of ASD.

## 1. Introduction

Autism spectrum disorder (ASD) is a neurodevelopmental disorder exhibiting a wide phenotypic scope and characterized by impairment in communication skills, social interaction, and behavior (restricted or repetitive) [1]. ASD is usually diagnosed during childhood, and mild autism is sometimes diagnosed only during adulthood [2,3]. It is an extremely heterogeneous disorder and could develop due to inheritable or de novo gene variations. Although hundreds of genes have been associated as contributors, in most cases the etiology remains unknown [4]. Thus, ASD is now assumed to be a disorder of complex interaction involving genetics, epigenetics, and the environment [5]. Common contributors to the development of the disorder include point mutations [6], copy number variants (CNVs) [7], translocations [8], DNA methylation [9], histone modifications [10], miRNAs expression [11], mitochondrial deficiencies [12], viral infections [13,14], aberrant gut microbiome composition [15], parental age, and environmental influences [16,17].

While understanding of the neurobiology and genetics of ASD has greatly improved in recent years, the diagnosis of ASD remains mainly based on defined behavioral and clinical symptoms reported by ASD children’s primary caregivers and clinicians’ assessment. Early diagnosis, ideally by age of 3–4 years, is crucial for starting behavioral therapy at an early age, which is critical for reducing ASD symptoms, strengthening communication skills, guiding parents, and improving ASD patients’ quality of life. Despite increasing awareness and monitoring of ASD rates for two decades, the average age of diagnosis, at least in the USA, has not improved [18]. This may be, in part, due to the fact that no unbiased systematic approach or medical test for the detection of ASD has been adopted into clinical practice. Indeed, although there are many biomarkers under development, most require replication and validation [19].

Machine learning (ML), sometimes referred to as “deep learning”, is a subfield of artificial intelligence (AI) research that analyzes data by computerized analytical model building. ML models are built based on statistical algorithms and are fitting for complex problem-solving involving multiple possibilities and combinations where conventional computational models might fail. Consequently, ML may provide tools to considerably increase the function of computational methods in neuroscience as well as improve clinical diagnosis and assist in the selection of treatment options. In recent years, considerable research has been applied in developing ML models to classify neuronal pathways and improve the understanding of mental disorders [20,21], Parkinson’s disease [22,23], Alzheimer’s disease [24,25], epilepsy [26], gestational diabetes [27], blood infections [28], COVID-19 [29], and more. Studies applying ML tools in ASD research include mainly models based on brain imaging data [30,31,32,33], but also behavioral evaluations [34,35,36,37], kinematic data [38,39], parental ages [40], eye movement data [41], and audio communication samples [42]. With ASD being a complex heterogeneous disorder, ML models based on genetic and/or genomic information are more limited. Recently published studies in this field focused on data retrieved from rare copy number variations (CNV) [43], long non-coding RNA (lncRNA) gene expression [44], and genome-wide association study (GWAS) meta-analysis [45]. ML models for ASD diagnosis were proposed mostly using DNA variant analysis [46,47]. Some ASD ML-based models examined RNA levels using in vitro cellular systems [48] or in silico data mining [49]. One study proposed a ML tool for ASD diagnosis based on salivary RNA [50]. However, blood samples are more readily available than saliva in toddlers. To our knowledge, no ML tools based on blood RNA expression levels have been reported for pediatric ASD diagnosis.

In this study, we aimed to generate predictive ML models for pediatric ASD diagnosis by utilizing our datasets of RNA expression levels in whole blood samples of children with ASD and neurotypical (NT) control children using quantitative real-time quantitative polymerase chain reaction (RT-qPCR) data. The RT-qPCR database applied for our current study is composed of the RNA expression levels of 10 studied genes found dysregulated in ASD and reported in our recently published research article, which was based on genome-wide RNA sequencing (RNA-seq) of peripheral blood samples from 73 American and Israeli children with ASD and 26 NT children [51]. Here, we present our ML-generated tool as a tentative proof-of-concept study that, once validated and improved using far larger cohorts of children with ASD and NT children, may potentially serve as an unbiased adjacent tool for early diagnosis of ASD.

## 2. Results

### 2.1. Choosing the Optimal Gene Combinations for ML Models

**Figure 1:**
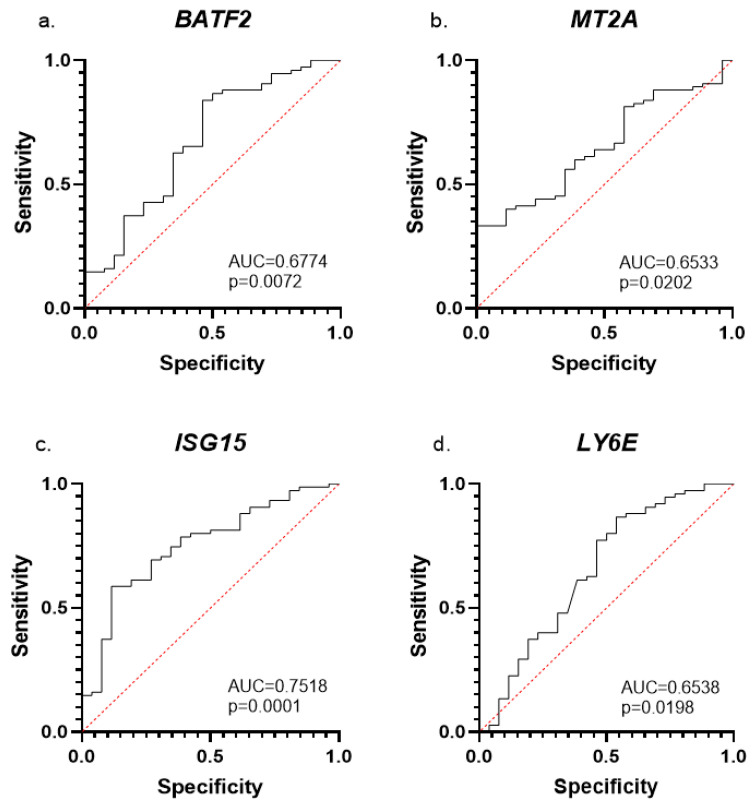
**ROC analysis of ASD-specific mRNA expression biomarkers**. AUC and *p*-values are displayed for (**a**) *BATF2*, (**b**) *MT2A*, (**c**) *ISG15,* and (**d**) *LY6E*.

We first evaluated the utility of a dataset of differently expressed genes in blood samples from ASD and NT individuals to serve as a potential ASD diagnostic tool. We performed ROC analysis with four genes that we recently reported as dysregulated in the blood of 73 children with ASD compared with 26 NT children [51]: *BATF2, LY6E, MT2A,* and *ISG15.* ROC analyses by mRNA expression with AUC > 0.5 for each of the tested genes alone (Figure 1): *BATF2* (AUC = 0.6774, *p* = 0. 0072), *MT2A* (AUC = 0.6553, *p* = 0.02), *ISG15* (AUC = 0.7518, *p* = 0.0001), and *LY6E* (AUC = 0.6538, *p* = 0.0198). These AUC and *p* values indicate a statistically significant distribution between the ASD and NT control groups. Pearson r correlation analysis was applied to the above four genes with diagnostic significance as detected by the ROC analysis for determining the gene combination with the highest predictive capacity based on our RT-qPCR data. The chosen gene combinations (predictors) for ML testing were those with correlations of r > 0.3 and *p ≤ 0.05: (#1) *BATF2, LY6E, MT2A,* and *ISG15;* (#2) *BATF2, SERPING1, MT2A,* and *FBXO*6; (#3) *MT2A, ISG15, FBXO6, SERPING1,* and *BATF2*; (#4) *MT2A, ISG15,* and *FBXO6* (shown in bold fonts in Supplementary Table S1). An additional fifth predictor was chosen based on the results of the random forest classifier feature importance [52]. The fifth predictor is a combination of the following five genes with the highest importance as shown by the classifier: *BATF2, ISG15, SERPING1, LY6E,* and *EFHC2* (Supplementary Figure S1).

### 2.2. Detection of an Optimized Diagnostic Model

**Figure 2:**
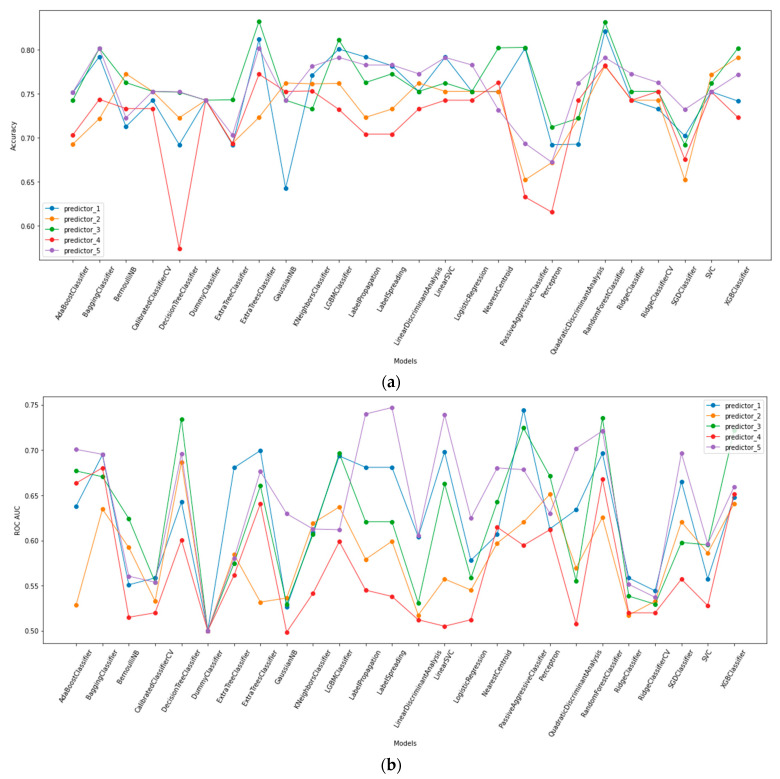
**Summary of Lazy Predict library results of 36 ML models for five established predictors.** (**a**) Accuracy score, (**b**) ROC AUC score. Two models were removed due to poor fit. Predictor_1: *BATF2, LY6E, MT2A* and *ISG15.* Predictor_2: *BATF2, SERPING1, MT2A,* and *FBXO*6. Predictor_3: *MT2A, ISG15, FBXO6, SERPING1,* and *BATF2*. Predictor_4: *MT2A, ISG15,* and *FBXO6.* Predictor_5: *BATF2*, *ISG15*, *SERPING1*, *LY6E,* and *EFHC2*.

**Figure 3:**
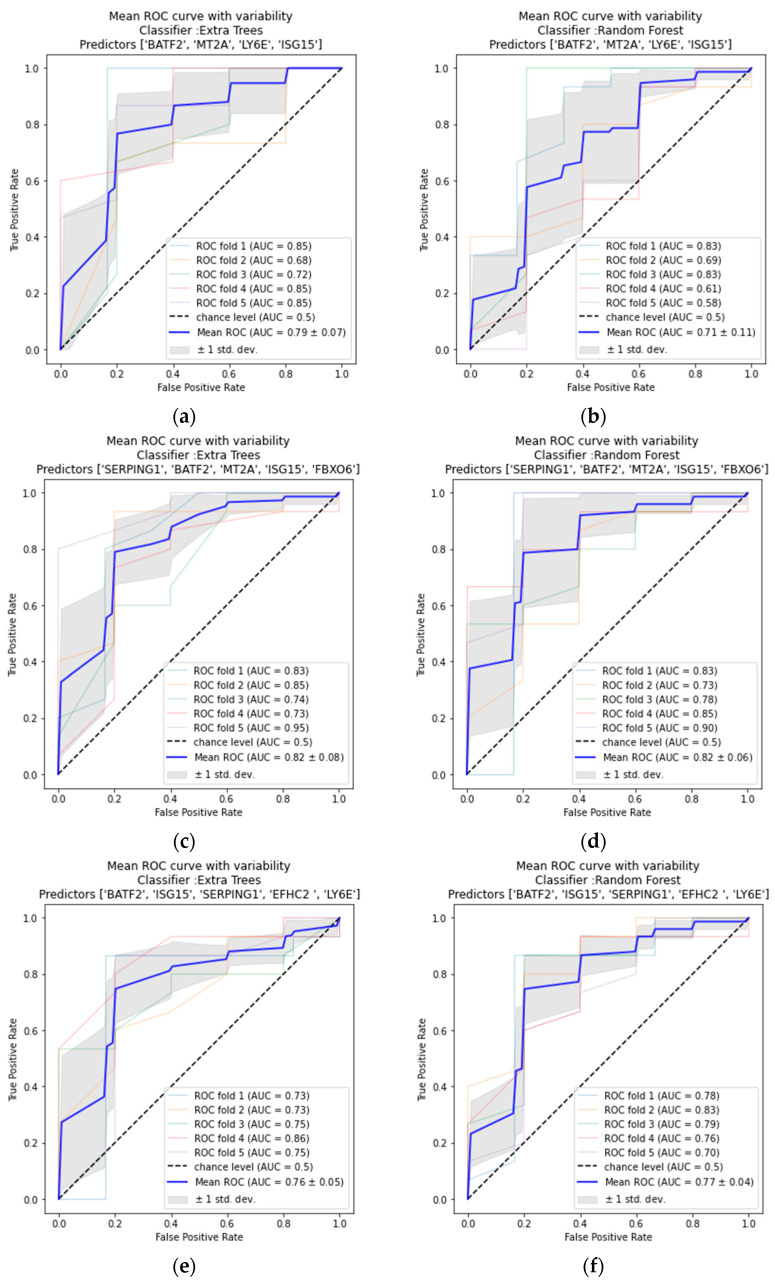
**Results of mean ROC curve with variability for different predictors: #1, #3 and #5, based on our ML models.** Predictor #1 (**a**) Extra Trees classifier, (**b**) Random Forest classifier. Predictor #3 (**c**) Extra Trees classifier, (**d**) Random Forest classifier. Predictor #5 (**e**) Extra Trees classifier, (**f**) Random Forest classifier. Each ROC AUC consists of a 5-StratifiedKFold average.

Based on our ROC analysis, Pearson r correlations values (Supplementary Table S1), and MDI feature importance evaluations, we chose the five mRNA expression combinations described above (Section 2.1). Next, we applied the Lazy Predict tool to the chosen predictors to determine which of the 36 ML models is most suitable for our randomized data sets. Extra trees and random forest classifiers presented the highest accuracy and ROC AUC values. Both ML models worked most accurately with predictors #1 (*BATF2*, *LY6E*, *MT2A* and *ISG15*), #3 (*MT2A*, *ISG15*, *FBXO6*, *SERPING1* and *BATF2*), and #5 (*BATF2*, *ISG15*, *SERPING1*, *LY6E* and *EFHC2*; Figure 2). To review the efficiency of extra trees and random forest classifiers, we measured the accuracy score using the leave-one-out cross-validator and performed ROC AUC measurements using stratified K-folds cross-validator. All calculations were made for the three chosen predictors (Table 1 and Figure 3). Results presented in Table 1 show the highest accuracy for predictors #3 and #5 when using the random forest classifier (accuracy = 82.178%; AUC = 0.82, 0.77, respectively). The combination consisting of the four significantly dysregulated and RT-qPCR validated genes in our recently published article (*BATF2, LY6E, MT2A,* and *ISG15;* predictor #1) also produced a highly accurate result when applying the extra trees classifier (accuracy = 81.188%, ROC AUC = 0.79; Table 1 and Figure 3).

## 3. Discussion

In this proof-of-concept study, we aimed to demonstrate the utility of blood transcriptomic data from small cohorts for building a ML-based tentative tool for distinguishing between children with ASD and NT children. Gene combination predictors were identified based on a combination of ML methods with RT-qPCR data generated blood gene expression values (2^^-ΔCt^) that yielded an accuracy of 82% in correctly identifying children with ASD and NT children. Two of our five ML models presented the highest suitability for our dataset: (1) *MT2A, ISG15, FBXO6, SERPING1,* and *BATF2*; (2) *BATF2*, *ISG15*, *SERPING1*, *LY6E,* and *EFHC2*. All the genes included in these two predictors have significance in ASD etiology, as discussed in our recent publication [51].

Our ML-generated tools described here should be considered as a proof-of-concept study and a preliminary guide for further studies on transcriptomics-based ASD diagnostics. Key limitations of the study include its small sample size and the possibility of model overfitting due to the absence of independent validation cohorts. In addition, the connection between the human peripheral blood and the brain transcriptomic profiles in individuals is poorly understood. Studies suggest that between 35% and 80% of known human transcripts are expressed in both the brain and blood, indicating a thoughtful use is needed when purposing peripheral gene expression as a proxy for gene expression in the CNS [53,54]. Therefore, the findings presented in this study should be interpreted carefully. Yet, keeping in mind that RT-qPCR studies (or custom-built gene expression microarrays) of blood samples are more accessible and affordable compared with brain MRI or fMRI scans, the diagnostic potential of ML-based tools for the detection of individuals with ASD following analysis of blood gene expression levels deserves further exploration. Analysis in larger cohorts should be carried out for improving and refining the ML tools proposed here. Future studies should also consider the influence of additional factors, including sex, age, ethnicity, and other confounders affecting associations between ASD phenotypes and blood genomic markers in their ML algorithms. This approach may eventually assist in the identification of a panel of biomarkers, leading to the earlier diagnosis of ASD among children with atypical neurodevelopment and to the stratification of the ASD population to different pathophysiologically relevant subgroups. Hence, RNA-based ML tools may provide better-personalized treatment alternatives for individuals with ASD.

## 4. Materials and Methods

### 4.1. Data Collection

The data used for this study are RNA expression levels of genes quantified by RT-qPCR (2^−ΔCt^). Data were obtained as described by Voinsky et al., 2022. Briefly, whole blood samples were collected from 73 ASD children and 26 NT controls in two cohorts (Israel and USA). RNA sequencing was performed on a subset of the samples. Next, the top 10 genes which were differentially expressed between the ASD and NT groups (p_adj_ < 0.05) were validated by RT-qPCR experiments, containing all samples in the Israeli and American cohorts. RNA expression levels of the following 10 genes were studied: *SERPING1*, *EFHC2*, *BATF2*, *CDC20*, *FCGR1A*, *MT2A*, *ISG15*, *FBXO6*, LINC00869, and *LY6E; GAPDH* was used as the qPCR control gene. The description of these genes, including their Gene ID codes, is provide in the work of Voinsky et al. [51]. All procedures and protocols were previously explained. Notably, of these 10 genes, two (*BATF2* and *LY6E*) were found upregulated and two (*ISG15* and *MT2A*) were found downregulated in blood samples from our combined American and Israeli cohorts of 73 children with ASD and 26 NT children [51].

### 4.2. Data Pre-Processing

Pre-processing of data was required for handling null values, missing in some samples due to removal in cases of a low quantity of tested samples. As such data were lacking at random, there is no specific structure to explain this absence, and missing values were replaced with median imputation [55,56]. Original and processed data were compared and found to present no significant statistical difference (*p* > 0.05).

### 4.3. Selecting Feature Importance

Random forest classifier was applied, working with the scikit-learn library [57] and using the RandomForestClassifier method (Python software v. 3.9), to determine the contribution of each of the 10 genes to the model prediction. Feature importance in the random forest classifier is based on a mean decrease in impurity (MDI). Thus, a score is computed based on the mean and standard deviation of accumulation of the impurity decline within each tree. In the scikit-learn library, for each decision tree, the library calculates an importance node using an MDI, with only two child nodes assumed (a binary tree):

(1) $$ni_{j}=w_{j}C_{j}-w_{left\left(j\right)}C_{left\left(j\right)}-w_{right\left(j\right)}C_{right\left(j\right)}$$

where *ni_j_* = node *j* importance, *w_j_* = weighted number of samples reaching node *j*, *C_j_* = node *j* impurity value, left (*j*)= child node from left split on node *j*, and right (*j*) = child node from right split on node *j*.

Next, the importance of each feature on a decision tree is determined as:

(2) $$fi_{i}=\frac{\sum_{j:node\;j\;splits\;on\;feature\;i}ni_{j}}{\sum_{k\in all\;nodes}ni_{k}}$$

where *fi _i_* = the importance of feature *I*, *n_ij_* = the importance of node *j*.

Later, this value can then be normalized by dividing by the sum of all feature importance values:

(3) $$normfi_{i}=\frac{fi_{i}}{\sum_{j\in all\;features}fi_{j}}$$

The random forest’s final feature importance is its average over all the trees. The total value of the feature’s importance on each tree is determined and then divided by the total number of trees:

(4) $$RFfi_{i}=\frac{\sum_{j\in all\;trees}normfi_{ij}}{T}$$

where *RFfi_i_* = the importance of feature *i* calculated from all trees in the random forest model, *normfi_ij_* = the normalized feature importance for *i* in tree j, and *T*= sum of trees (total). For the predictor combination, we used genes with an MDI score > 1. Additionally, we focused on the four dysregulated genes from our previously published study that were validated by RT-qPCR in the combined American and Israeli cohorts [51]: *BATF2*, *LY6E*, *MT2A,* and *ISG15*. To evaluate the dysregulated genes’ diagnostic value, ROC (receiver operating characteristic) analysis was utilized [58]. Next, Spearman r correlation analysis was used for each dysregulated gene with other significantly differentially expressed genes (as defined in “Section 4.1. Data Collection”). For the correlation test, *p* ≤  0.05 was considered significant. ROC and Spearman analyses were performed using GraphPad Prism v. 9 software (San Diego, CA, USA).

### 4.4. Machine Learning Algorithms

The Lazy Predict python library (https://lazypredict.readthedocs.io/en/latest/, accessed on 1 September 2022) was used to evaluate the most applicable ML algorithms for the prediction of ASD transcriptomic signature. Lazy Predict is a library that builds 36 basic ML models, suggesting the most suitable model for prediction variables prior to testing against hyperparameters. Consequently, two ML models were selected for further inspection, random forest classifier and the extra trees (extremely randomized trees) classifier. Random forest is a controlled ensemble learning algorithm that consists of many small decision trees (estimators), each generating its own prediction [59]. The random forest algorithm creates and combines multiple decision trees into one “forest” to deliver a more accurate prediction. Extra trees classifier is also an ensemble learning algorithm, like random forest, except for the random selection of split values in the data [60]. That is, while random forest selects cut points to split connections at an optimal split, extra trees chooses them randomly. Next, we applied grid search, an optimization tool used to select the best combination of parameters, for tuning the hyperparameters in our models. The chosen models and tools were applied using the scikit-learn methods RandomForestClassifier, ExtraTreesClassifier, and GridSearchCV. All the methods were computed using Python software v. 3.9.

### 4.5. Accuracy and ROC AUC Validation

For validating the accuracy of our ML algorithms, we applied the leave-one-out cross-validator, using the scikit-learn method LeaveOneOut. This method is favored when analyzing small data sets, such as the one we used for this study. In this form of validation, the number of folds equals the number of cases in the data set. Hence, it uses a selected case as a single-item test set, where the learning algorithm is applied once for each case, and all other cases are used as a training set. To assess our models, we visualized the variance of the ROC metrics using cross-validation. Scikit-learn library methods were utilized. The RocCurveDisplay method was used to draw the curves, StratifiedKFold method computed the fold groups, and the auc method was used to calculate the area under the curve (AUC) using the trapezoidal rule. All the methods were computed using Python software v. 3.9.

## Figures and Tables

**Table 1:** **Summary of model accuracy results for different gene combinations (predictors), based on RT-qPCR results (2^−ΔCt^)**: (#1) *BATF2, LY6E, MT2A* and *ISG15*, (#3) *MT2A, ISG15, FBXO6, SERPING1*, and *BATF2* (#5) *BATF2*, *ISG15*, *SERPING1*, *LY6E*, and *EFHC2*.

	Predictor	#1	#3	#5
Model Accuracy				
*Extra Trees Classifier*		**81.188%**	80.198%	80.198%
*Random Forest Classifier*		79.208%	**82.178%**	**82.178%**

## Data Availability

The datasets and code generated for the current study, including the real-time qPCR spreadsheets, are available from I.V. or D.G. on reasonable request.

## Supplementary Materials

The following supporting information can be downloaded at: https://www.mdpi.com/article/10.3390/ijms24032082/s1.
