# Supplementary material for: Machine Learning-Based Blood RNA Signature for Diagnosis of Autism Spectrum Disorder
Source: Int J Mol Sci. 2023 Jan 20;24(3):2082. doi: 10.3390/ijms24032082 (PMC9916487; doi:10.3390/ijms24032082)

Supplementary materials for:

# Machine Learning-Based Blood RNA Signature for Diagnosis of Autism Spectrum Disorder

**Irena Voinsky [1], Oleg Y. Fridland [2], Adi Aran [3,4], Richard E. Frye [5,6,*] and David Gurwitz [1,7,*]**

[1] Department of Human Molecular Genetics and Biochemistry, Faculty of Medicine, Tel Aviv University, Tel Aviv 69978, Israel; irenavoinsky@mail.tau.ac.il
[2] Independent Researcher, Tel Aviv 69978, Israel; olegfrid@post.bgu.ac.il
[3] Shaare Zedek Medical Center, Jerusalem 91031, Israel; aaran@szmc.org.il
[4] Obesity and Metabolism Laboratory, Institute for Drug Research, School of Pharmacy, Faculty of Medicine, The Hebrew University of Jerusalem, Jerusalem 91240, Israel
[5] Autism Discovery and Treatment Foundation, Phoenix, AZ 85050, USA
[6] Rossignol Medical Center, Phoenix, AZ 85050, USA
[7] Sagol School of Neuroscience, Tel Aviv University, Tel Aviv 69978, Israel
* Correspondence: drfrye@rossignolmedicalcenter.com or drfrye@autismdiscovery.org (R.E.F.); gurwitz@tauex.tau.ac.il (D.G.)

**Supplementary Table S1: Pearson r correlation of studied genes expression (2^$^{--\Delta Ct}$).** The number of XY Pairs=101. Correlation in bold represent chosen genes combinations for ML testing (r>0.3, *p≤0.05). *P≤0.05, **P≤0.01, ***P≤0.001, ****P≤0.0001.

| Gene | *BATF2* | *MT2A* | *ISG15* | *LY6E* |
|---|---|---|---|---|
| *SERPING1* | **r=0.3030<br>**p=0.0021** | **r=0.4525<br>****p<0.0001** | r=0.2948<br>**p=0.0028 | r=-0.1348<br>p=0.1791 |
| *BATF2* | | **r=0.3303<br>***p=0.0007** | r=0.09726<br>p=0.3332 | r=0.2832<br>**p=0.0041 |
| *EFHC2* | r=-0.006877<br>p=0.9456 | r=-0.1668<br>p=0.0956 | r=0.007879<br>p=0.9377 | r=-0.1816<br>p=0.0692 |
| *FCGR1A* | r=0.2766<br>**p=0.0051 | r=0.1139<br>p=0.2567 | r=0.2377<br>*p=0.0167 | r=-0.1737<br>p=0.0824 |
| *CDC20* | r=0.2495<br>*p=0.0119 | r=0.02850<br>p=0.7772 | r=0.09243<br>p=0.3579 | r=-0.1396<br>p=0.1639 |
| *MT2A* | **r=0.3303<br>***p =0.0007** | | **r=0.3126<br>**p=0.0015** | r=0.009802<br>p=0.9225 |
| *ISG15* | r=-0.006877<br>p=0.9456 | **r=0.3126<br>**p=0.0015** | | r=0.1379<br>p=0.1692 |
| *FBXO6* | **r=0.3134<br>**p=0.0014** | **r=0.3884<br>****p<0.0001** | **r=0.3618<br>***p=0.0002** | **r=0.4343<br>****p<0.0001** |
| *LINC00869* | r=-0.03090<br>p=0.7590 | r=0.1456<br>p=0.1463 | r=0.1852<br>p=0.0637 | r=0.006298<br>p=0.9502 |
| *LY6E* | r=0.2832 | r=0.009802 | r=0.1379 | |

| | | | | |
|---|---|---|---|---|
| | **p=0.0041 | p=0.9225 | p=0.1692 | |

**Supplementary Figure S1: Feature importance using the Random Forest Classifier.** The bars represent the feature importance of the “forest”, and standard deviation error bars represent the inter-trees variability. See Methods 2.3 for further details.

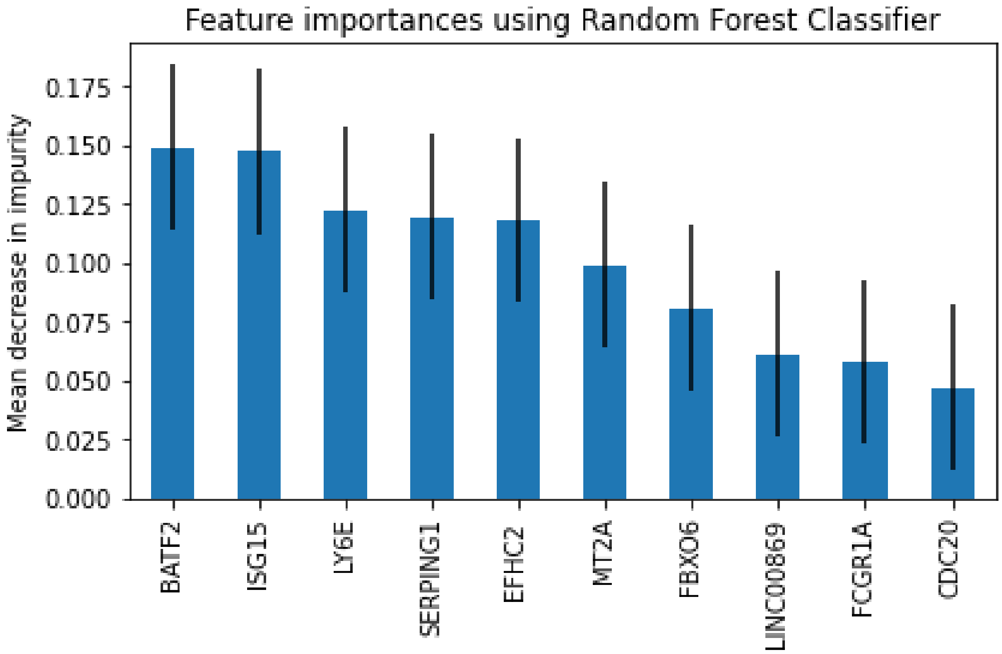

Supplement: Supplementary file 1 [file ijms-24-02082-s001.zip › ijms-2131524-supplementary.pdf]
